# Regional activity and interregional functional connectivity uniquely contribute to social cognitive judgments during movie-watching

**DOI:** 10.1162/NETN.a.571

**Published:** 2026-07-27

**Authors:** Roberto C. French, Haily Merritt, Colleen Hughes, Richard Betzel, Anne C. Krendl

**Affiliations:** Department of Psychological and Brain Sciences, Indiana University, Bloomington, IN, USA; Department of Informatics & Program in Cognitive Science, Indiana University, Bloomington, IN, USA; Department of Neuroscience, University of Minnesota, Minneapolis, MN, USA; Masonic Institute for the Developing Brain, University of Minnesota, Minneapolis, MN, USA

**Keywords:** fMRI, Social cognition, Dynamic functional connectivity, Brain-behavior relationship, Movie watching

## Abstract

Understanding how the brain gives rise to social cognition has been a key goal of neuroimaging research. Both changes in regional activation as well as functional connectivity have been implicated as potential mechanisms underlying social cognition, but the two have rarely been examined concurrently. Moreover, because the neural processes underlying social cognition are dynamic, developing approaches to capture dynamic changes in regional activity and functional connectivity are critical. Here, we describe a novel analysis approach that captures both regional activity and dynamic functional connectivity simultaneously during a naturalistic, socially focused movie-watching task. We found that both regional activation and functional connectivity were uniquely related to awkwardness, a judgment associated with social faux pas detection and theory of mind. Regional activation within sensorimotor networks was positively associated with awkwardness, whereas activation in the default network was negatively associated. Models including functional connectivity accounted for unique variance beyond models with activity alone. Specifically, dynamic functional connectivity between networks, primarily the frontoparietal control network, was positively associated with awkwardness. Together, these findings suggest that both dynamic regional brain activity and functional connectivity each uniquely contribute to complex and dynamic social judgments.

## Author Summary

We assessed the relationship between regional activity, functional connectivity, and awkwardness judgments during a video-watching task using fMRI in a cohort of healthy young adults. We used a novel analysis framework to simultaneously disentangle the unique contributions of dynamic regional activity and dynamic functional connectivity in their predictions of awkwardness. We found regional activity associated with awkwardness primarily in sensorimotor, frontoparietal control, and default networks, and functional connectivity associated with awkwardness within and between the visual, dorsal attention, frontoparietal control, and default networks. Finally, hierarchical models demonstrated that modeling functional connectivity significantly explained additional variance beyond modeling the activity of two regions alone, indicating the unique explanatory power offered by dynamic functional connectivity in relation to behavior.

## INTRODUCTION

Social cognition, the manner in which we process, store, and remember information about others (Adolphs, 1999; Sherman et al., 1989), plays a key role in navigating social interactions. Though the neural correlates underlying social cognition have been widely studied (Frith & Frith, 2012; Green et al., 2019; Lieberman, 2007; Mar, 2011; Schurz et al., 2021; Van Overwalle, 2009; Wittmann et al., 2018), the preponderance of this work has focused on either localizing patterns of BOLD activity (regional brain activation) (Arioli et al., 2021) or examining changes in functional connectivity—correlations in brain activity between regions—in response to social *versus* nonsocial processes (Maliske & Kanske, 2022; Maliske, Lehmann, et al., 2023; Maliske, Schurz, & Kanske, 2023). However, there are two key limitations to this work. First, it has largely overlooked the dynamic neural processes underlying social cognition. This is important because social interactions in everyday life unfold over prolonged timescales and are informed by multiple rich channels of information (i.e., they are complex) (Tamir & Thornton, 2024). Thus, dynamic functional connectivity may provide greater insight into the cognitive processes and fluctuations that underlie dynamic social judgments (Krendl & Betzel, 2022). Second, because dynamic functional connectivity and regional activity have been examined separately, it is unclear to what degree dynamic functional connectivity uniquely contributes to social cognitive processing beyond regional activity. In the current study, we attempt to disentangle these related neural processes by examining them concurrently and in a dynamic task.

Although brain connectivity and activity are often assessed separately as independent measures, they are closely related. Despite this, there is limited work that accounts for the potential overlapping BOLD signal between regional activity and connectivity. This is important as functional connectivity has been shown to predict trait-level social cognition (Christov-Moore et al., 2020; Markett et al., 2018), though much of this work assesses static functional connectivity during rest. Both regional brain activity and functional connectivity are dynamic processes (Esfahlani et al., 2022; Hasson et al., 2008; Nastase et al., 2019; Sporns et al., 2021) that vary temporally in association with cognitive demands (Friston et al., 1994). Prior work using dynamic functional connectivity approaches has shown that functional brain networks are more integrated during cognitive tasks (Shine et al., 2016), that transitions to metastable brain states are related to external cognitive function (Nomi et al., 2017), and has found distinct patterns in response to dissociable tasks (Chen et al., 2017; Cohen, 2018). These patterns may therefore capture unique information about how brain systems reorganize during cognitive processes that cannot be explained by regional activity alone.

Studying regional activity and functional connectivity separately has limitations. For example, prior work suggests that head motion (Laumann et al., 2017; Lurie et al., 2020) and task activations (Al-Aidroos et al., 2012; Cole et al., 2019, 2021) can spuriously inflate functional connectivity estimates. Thus, functional connectivity analyses on task-based neuroimaging designs have controlled for task activations via explicit modeling (Cole et al., 2019, 2021), which elicits more stable results than resting state measures alone (Gonzalez-Castillo & Bandettini, 2018; Gonzalez-Castillo et al., 2015), or through careful experimental design (Rissman et al., 2004). With respect to regional activity, there are few frameworks that attempt to control for connectivity within activation studies (but see work using psychophysiological interaction analysis [Friston et al., 1997]), making it challenging to disentangle regional activity from functional activity.

A simultaneous model of regional activity and functional connectivity associated with behavior bridges the gap between both of these issues, and may better model complex cognitive and behavioral states, such as those engaged during dynamic social judgments (Wolf et al., 2010). Edge time series, which reflect the dynamic co-fluctuations between pairs of brain regions, provides an analytical framework for doing this (Merritt et al., 2025; Tanner et al., 2024). Critically, a recent extension of this work validated a method to model dynamic activity alongside dynamic connectivity in zebrafish and human functional magnetic resonance imaging (fMRI) data (Merritt et al., 2025). We applied this model in the current study to identify where dynamic regional activity, and their interacting functional connectivity, predicted dynamic social cognitive judgments such as those engaged in naturalistic (e.g., movie-watching) paradigms.

Movie-watching paradigms are well suited to model the relationships between dynamic functional connectivity and regional activity (Lurie et al., 2020) because they allow for the representation and observation of temporally unfolding and dynamic social interactions similar to those encountered in everyday life (Jääskeläinen et al., 2021; Sonkusare et al., 2019; Zhang et al., 2021). In addition to having greater ecological validity than static tasks (Nastase et al., 2020; Osborne-Crowley, 2020), movie-watching is more sensitive to detecting cognitive decline and clinical impairment than static experimental procedures (Chumin et al., 2024; Cutts et al., 2025). The current study thus used a novel network neuroscience approach to disentangle the unique contributions of dynamic regional brain activity from dynamic functional connectivity (Faskowitz et al., 2020; Merritt et al., 2025; Zamani Esfahlani et al., 2020) in predicting dynamic social cognitive judgments, specifically theory of mind—the ability to infer others’ thoughts and emotions (Frith & Frith, 2005).

We examined constructs related to theory of mind because theory of mind is a complex, multifaceted, and dynamic process (Tamir & Thornton, 2024) that is disrupted in many clinical and aging populations (Bishop-Fitzpatrick et al., 2017; Bora et al., 2009; Henry et al., 2013). An important benefit of focusing on theory of mind-related judgments in the current task is that its associated neural correlates have been well-characterized (Schurz et al., 2021). However, this work typically examines theory of mind statically (e.g., averaged over the entire task; Schurz et al., 2021), rather than dynamically (but see Lee Masson et al., 2024; Moran et al., 2004; Pantelis et al., 2015). A static examination of theory of mind may obfuscate the mechanisms underlying temporally unfolding aspects of theory of mind processing. Regardless, a key finding in this work is that theory of mind is supported by a distributed system in the midline of the brain, including the medial prefrontal cortex, temporoparietal junction, and precuneus, which play unique, but complementary, roles during mentalizing processes (Schurz et al., 2021). For example, the medial prefrontal cortex represents knowledge about the self and others (Wagner et al., 2012), whereas the temporoparietal junction dissociates knowledge about the self from knowledge about others (Quesque & Brass, 2019). The precuneus, however, supports both theory of mind and autobiographical memory (Rabin et al., 2010; Spreng et al., 2009), which allows individuals to remember and draw from prior experiences when inferring others’ mental states, (e.g., Perner et al., 2007).

An important limitation of widely used dynamic measures of theory of mind is that they often require participants to explicitly engage in theory of mind (e.g., by asking theory of mind-related questions after presenting a series of short clips; e.g., the Awareness of Social Inference Test (TASIT), the Movie for the Assessment of Social Cognition (MASC); Dziobek et al., 2006; McDonald et al., 2006). This approach reduces the task’s ecological validity by removing the spontaneous nature of social cognitive judgments (Tamir & Thornton, 2024). An alternative approach that has been used in prior work involves having participants passively view dynamic movies while undergoing scanning (e.g., Pantelis et al., 2015; Przysinda et al., 2024). For example, one study had participants passively view an episode of the television show, *The Office*, while undergoing fMRI; a separate group of participants viewed the same episode outside of the scanner while making continuous ratings of “social awkwardness” (Pantelis et al., 2015). The authors selected awkwardness because it is a core feature of theory of mind (Baron-Cohen et al., 1999a, 1999b).

Detecting awkwardness is analogous to the widely studied ability to detect social norm violations or socially inappropriate behavior (Heavey et al., 2000) because it requires that participants understand the mental and/or emotional states of multiple characters (e.g., that one character’s behaviors are not in line with another character’s expectations) as well as the context of the situation (e.g., behavior appropriate among friends may not be appropriate with strangers). Consistent with that framing, the study and another (Przysinda et al., 2024) found that, when modeling awkwardness as a parametric regressor, spontaneously detecting awkwardness was associated with brain activity in core theory of mind regions (e.g., medial prefrontal cortex, precuneus, temporoparietal junction). Moreover, participants had high consensus about which moments of the episode were socially awkward (Pantelis et al., 2015). This finding indicates that awkwardness is an accessible operationalization of theory of mind because it has shared (vs. idiosyncratic) understanding across participants. Additionally, continuous ratings of awkwardness predict independent assessments of theory of mind, both behaviorally and neurally (French et al., 2024). As such, using awkwardness as the operationalization of theory of mind thus holds several advantages; it both theoretically and empirically demonstrates overlap with theory of mind, is a valid construct that can be assessed spontaneously moment-to-moment, and elicits high consensus.

In the current study, we used a naturalistic viewing paradigm to disentangle the unique contributions of regional brain activity (node) and functional connectivity (edge) to the dynamic detection of awkwardness. Our conceptualization of social awkwardness aligned with related work (Pantelis et al., 2015). Specifically, it refers to situations in which someone, either intentionally or unintentionally, commits a social faux pas (Baron-Cohen et al., 1999a) or violates a social norm (Berthoz et al., 2002) and the ensuing uncomfortable response this elicits for those around them. By identifying unique activation and/or connectivity that predicted awkwardness, we could therefore more directly connect regional activation and functional connectivity to social cognition. It is important to note, however, that our goal was not to dissociate awkwardness from highly related psychological experiences, such as arousal (Singh & Bhushan, 2025). Because perceptual, affective, and attentional processes are deeply intertwined during real-world social experiences of social cognition (e.g., Redcay & Moraczewski, 2020), we therefore did not disentangle related, but potentially distinct, constructs (e.g., loudness, arousal) from awkwardness. We hypothesized that both activity and connectivity of overlapping and distinct brain regions would explain unique variance associated with awkwardness during movie-watching. The current study, therefore, presents a framework for testing and validating a novel method that disentangles the unique contributions of dynamic regional activity and functional connectivity to complex behavior.

## MATERIALS AND METHODS

### Demographics

As part of a larger study assessing social cognition, we recruited a young adult community sample from Bloomington, Indiana, who underwent fMRI in exchange for monetary compensation (*N* = 123; age range: 18–35 years). Participants were excluded from imaging analyses if they did not complete both movie-watching runs (13 participants) or if either of those runs had poor image quality (see Image Quality Assessment section, four participants). Data collection was approved by the Indiana University Institutional Review Board (#11801). The analyzed sample comprised 106 participants (see Table 1 for sample demographics).

**Table 1.** Demographic information on participants.

*N*	106
Age, years	
Mean (*SD*)	21.71 (4.21)
Range	18–35
Gender, count (%)	
Male	39 (36.8)
Female	64 (60.4)
Non-binary	3 (2.8)
Race, count (%)	
White	78 (73.6)
Black	1 (0.9)
Asian	17 (16)
Multiracial	10 (9.4)
Mean framewise displacement (mm)	
Movie 1 - Petting Zoo	0.14
Movie 2 - Souvenir Shop	0.13
Percent of frames exceeding 0.5-mm framewise displacement	
Movie 1 - Petting Zoo	1.51%
Movie 2 - Souvenir Shop	1.62%

### Movie-Watching Task

While undergoing fMRI, participants viewed two episodes: Season 1, Episode 2 (“Petting Zoo) and Season 2, Episode 2 (“Souvenir Shop”) of the mockumentary-style television show *Nathan for You*, which was specifically chosen to elicit moments of awkwardness during social interactions between the characters (for more information, see French et al., 2024). Participants were instructed to watch the shows as if they were viewing them at home. Each video was approximately 8 min long and presented via MATLAB (Version R2022b) utilizing the Psychophysics Toolbox Version 3 (Kleiner et al., 2007).

### Continuous Awkwardness Rating Task

**Figure 1.**
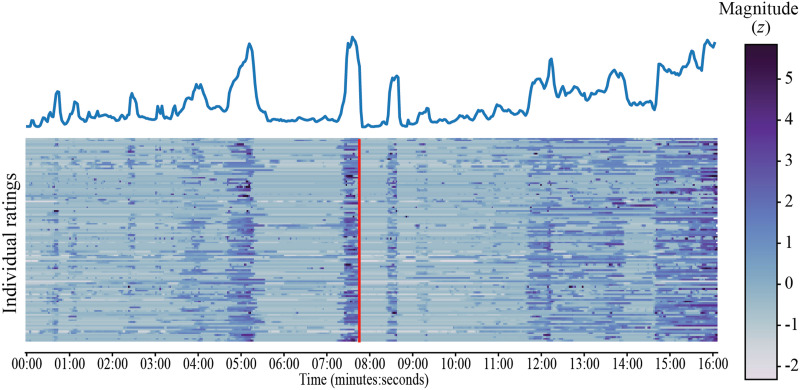
Depiction of awkwardness ratings from all individual ratings of the independent sample of raters (each row = one participant’s time series) over the course of the two videos. The top line depicts the average (consensus) rating of the individuals that was used in further analyses. The red line indicates the split between movies.

We assessed dynamic detection of awkwardness using a continuous rating task collected from an independent sample of undergraduates at Indiana University (*N* = 110; *M*_*Age*_ = 18.7 years; *SD* = 0.95) who participated in exchange for partial course credit. While watching the same two episodes of *Nathan for You*, participants made continuous ratings about the show’s awkwardness using a Logitech Extreme 3D Pro joystick. Participants were given no explicit definition of awkwardness. Rather, they were instructed to rely on their personal judgements of what they constituted as being awkward when moving the joystick. Ratings were sampled at approximately 30 Hz mirroring the frame rate of the video. Within individuals, ratings across the episodes were concatenated in the order of acquisition and *z* scored. Then, to derive a consensus, the ratings from all individuals were averaged into a single time series (see Figure 1). This procedure resulted in a continuous measurement of awkwardness. To assess the interrater reliability of the awkwardness consensus score, we employed a calculation of the intraclass correlation coefficient ICC(3, k) (Shrout & Fleiss, 1979). The ICC value for the set of consensus raters was 0.98, a high reliability measure; for further information on construct validity, see French et al. (2024).

### Image Acquisition

Neuroimaging was performed with a 20-channel head/neck coil on a Siemens 3.0T Prisma MRI Scanner at the Indiana University Imaging Research Facility in Bloomington, Indiana. The movies were presented using a projector illuminating a screen that was visible to participants through a mirror attached to the head coil. Anatomical scans were acquired with a high-resolution 3D magnetization prepared rapid gradient echo sequence (sagittal rotation; 160 slices, echo time (TE) = 2.7 ms, repetition time (TR) = 1,800 ms, inversion time (TI) = 900 ms, flip angle = 9°, 1.0-mm isotropic voxels; with no fat suppression). Functional scans were collected using an echo-planar image (EPI) sequence sensitive to BOLD contrast (T2*; 54 slices with 2.2 mm thickness and no gap, TE = 30 ms, TR = 2,000 ms, flip angle = 70°, 2.2-mm isotropic voxels, field of view (FOV) = 242 × 211.2 mm, in-plane matrix size = 110 × 96, anterior-to-posterior (A/P) phase encoding direction). Slices were collected in an interleaved order (multiband acceleration factor = 2). Prior to all functional imaging, phase-encoding polarity reversed single-echo EPI image pairs were acquired for distortion correction (54 slices with 2.2 mm thickness and no gap, TE = 58 ms, TR = 7,700 ms, flip angle = 90°, 2.2-mm isotropic voxels, FOV = 242 × 211.2 mm, in-plane matrix size = 110 × 96).

### Image Preprocessing

Initial anatomical and functional image preprocessing was performed using fMRIPrep Version 23.2.0 (Esteban et al., 2019) (RRID: SCR_016216), which is based on Nipype 1.8.6 (Gorgolewski et al., 2011) (RRID: SCR_002502). Detailed preprocessing is reported in the Supporting Information. In brief, functional images were realigned to correct for motion, underwent slice-timing correction, underwent distortion correction based on two EPI references, and were normalized to the MNI152NLin6Asym (Evans et al., 2012) template space. All resamplings were performed with a single interpolation step by composing all the pertinent transformations. Subsequent denoising appropriate for functional connectivity analyses was conducted using the eXtensible Connectivity Pipeline-DCAN (XCP-D) (Ciric et al., 2018; Satterthwaite et al., 2013). The first two volumes (4 s) were discarded from each run to correct for potential scanner inhomogeneities. Nuisance regressors included the fMRIPrep-calculated confounding time series for the three region-wise global signals extracted within the cerebrospinal fluid, white matter, and global masks, six head motion parameters, the temporal derivatives and quadratic terms for those nine terms, and discrete cosine regressors (analogous to a high-pass filter at 0.008 Hz), and linear trend and intercept terms. Volumes with framewise displacement greater than 0.5 mm were replaced using cubic spline interpolation implemented by XCP-D to preserve the intact time series. After denoising, the 200-region Schaefer parcellation (Schaefer et al., 2018) was applied to the residual BOLD signal. Parcellated time series for the two movie scans were then concatenated in the order of acquisition.

### Image Quality Assessment

fMRIPrep reports were manually inspected. One participant was excluded because they failed segmentation during fMRIPrep. Runs were flagged for exclusion if the mean framewise displacement across the full timeseries, without censoring, exceeded 1.5 times the interquartile range in the adverse direction. If any of the runs were flagged, that participant was excluded from analysis (*n* = 4). See Table 1 for descriptive statistics about motion among the analyzed sample.

## ANALYTIC APPROACH

### Individual-Level Analysis: Edge Time Series

**Figure 2.**
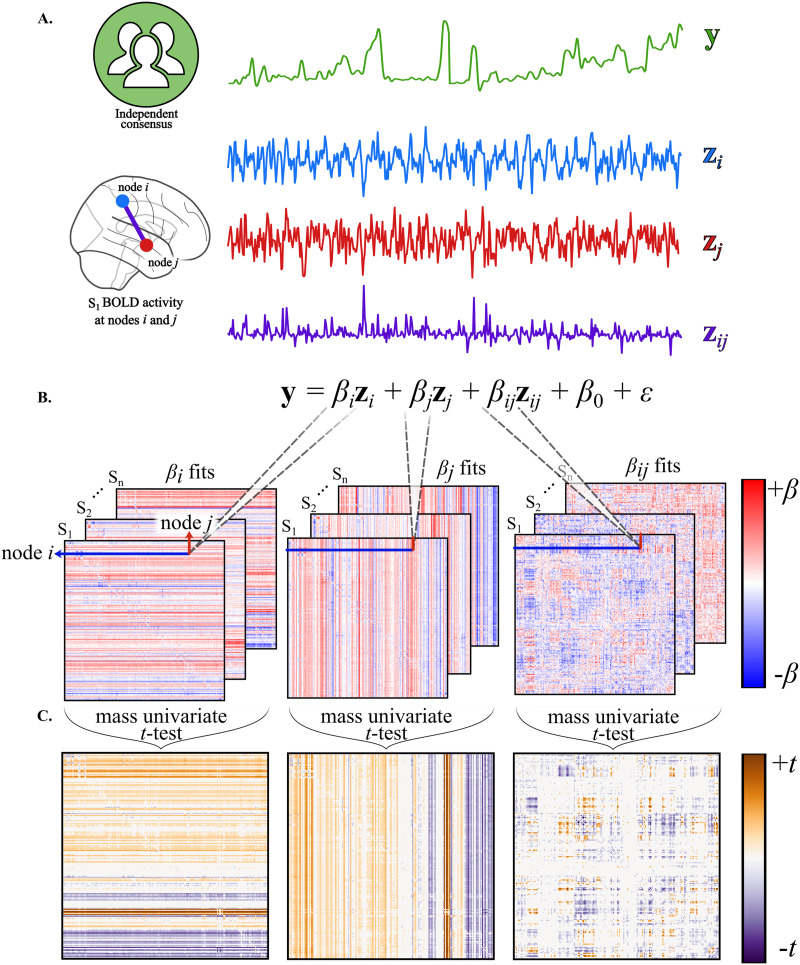
Representative schematic of analysis pipeline. (A) Independent consensus rating of awkwardness (green) alongside standardized nodal timeseries from node *i* (blue), node *j* (red), and their momentary co-fluctuation edge time series (purple) for example subject “S_1_”. (B) Linear model constructed across each pair of regions for every subject and the example subject level result matrices, depicting beta weights for each model term: node *i* (left), node *j* (center), and the edge time series *ij* (right). (C) Depiction of group-level results for each model term generated via mass univariate *t* tests from individual subject matrices presented in B.

Edge time series, which captures dynamic co-fluctuations between pairs of brain regions, were calculated as the element-wise product of the standardized signals of two nodes (brain regions) (see Figure 2A). This is mathematically equivalent to the commonly utilized product-moment correlation to calculate functional connectivity but omits the final averaging step that results in a single time-invariant value. In doing so, the procedure retains a time-resolved measure of the momentary co-fluctuation between pairs of nodes, the temporal resolution of which is equal to the temporal resolution of acquisition (2-s TR, in this case). For more information, see Faskowitz et al. (2020) and Zamani Esfahlani et al. (2020). We calculated the edge time series across all unique pairs of parcellated nodal time series (200 × 200 nodes), resulting in 19,900 edge time series.

### Individual-Level Analysis: Linear Model Construction

Following Merritt et al. (2025), we analyzed the contributions of nodes and edges to behavior for each participant individually. Specifically, we fit the following linear model for each pair of nodes, *i* and *j: y* = *β_i_z_i_* + *β_j_z_j_* + *β_ij_z_i_z_j_* + *β*_0_ + *ϵ*. Where *y* is the consensus awkwardness rating, *z*_*i*_ the standardized activity time series for node *i, z*_*j*_ the standardized activity time series for node *j*, and *z_i_z_j_* the edge time series between nodes *i* and *j* (see Figure 2B). Importantly, the edge time series is equivalent to the statistical interaction term in the linear model. Prior to model fitting, the awkwardness consensus was convolved with the Glover (Glover, 1999) hemodynamic response function via the “compute_regressor” function (Nilearn) (RRID:SCR_001362) to better represent the expected BOLD response.

Due to the high degree of serial autocorrelation within both the behavioral signal and the BOLD signal, we also performed prewhitening prior to estimating the model parameters *β_i_, β_j_, β_ij_*, and *β*_0_ (Merritt et al., 2025). Briefly, we calculated the residuals from the linear model fit, then fit an autoregressive model of order 25 to the residuals and retained the autoregressive coefficients. The coefficients are used to construct the prewhitening matrix **W**, which is multiplied by each term in a new regression model: *Wy* = *WXβ* + *Wϵ* and subsequently fit. Where *y* is the same behavior of interest, **X** is the matrix [*z*_*i*_, *z*_*j*_, *z*_*i*_*z_j_*, 1], and *β* the new regression coefficients. Using the statsmodels Python package (Seabold & Perktold, 2010), we fit autoregressive models using the AutoReg function and ordinary least squares linear regressions using the OLS function.

Fitting the linear model to all pairs of nodes resulted in a node × node matrix for each regression coefficient, *β_i_, β_j_*, and *β_ij_*. For clarity, we present only a single nodal matrix as *β_i_* and *β_j_* are transpositions of one another. These matrices represent the strength of relationship between each term and the awkwardness rating.

### Group-Level Analysis: Network-Level Significance Testing

We initially estimated matrices of regression coefficients, *β_i_, β_j_*, and *β_ij_*, at the level of individual participants. A one-sample *t* test (vs. zero; i.e., no association with behavior) was conducted across participants at each edge (node pair). This resulted in a single group-level matrix reflecting the association with behavior of each term (see Figure 2C). Given the large number of hypothesis tests performed, the group-level output matrices were thresholded to only retain weights that survived below *q* = 0.05 false discovery rate (FDR) correction. See Figure 4 for a graphical representation of the analytic approach.

The resulting matrices were then assessed for network-level significance, using a spin-test permutation (Váša et al., 2018). The spin test allows for permutation testing to break the association of edges to specific networks while retaining the spatial contiguity of nodes. A null distribution was created for each of the 17 network (Schaefer et al., 2018; Yeo et al., 2011), as well as the network-network off-diagonal blocks. The null distribution retained the count of suprathreshold edges within each block across 10,000 permutations. A network-level block was deemed significant if the true count of suprathreshold edges within the network fell below the *q* = 0.05 threshold after FDR correction for multiple comparisons. Spin tests were performed across suprathreshold positive and negative edges separately because they lead to different interpretations of the relationship with awkwardness. All data and code used for analyses can be found publicly available on OSF: https://osf.io/8bvu9/ and GitHub: https://github.com/rcf004/edge-timeseries-linear-interaction.

## RESULTS

### Node Activity Associated With Awkwardness

We first examined nodes whose activity was associated with awkwardness. To do this, we extracted the significant model results from the group-level statistical model for the activity term. Across the entire matrix of results, the nodal activity results were largely invariant across a single axis, hence the banding present in rows of Figure 2A. This can be explained as the fitted beta weight for any node *i* is not prone to much change in fit given any pair node *j* included in the model; for example, the relationship between awkwardness and insula activity (as node *i*) is largely the same regardless of whether medial prefrontal cortex or temporal pole were included in the model as node *j*. Given this invariance, we calculated the mean regression coefficient for each node (Supporting Information Table S1) and projected it to a brain surface (Figure 2B) to indicate the spatial location of nodes. Lastly, we visualized the network-level aggregation of nodal results (Figure 2C) for ease of comparison to the connectivity results.

**Figure 3.**
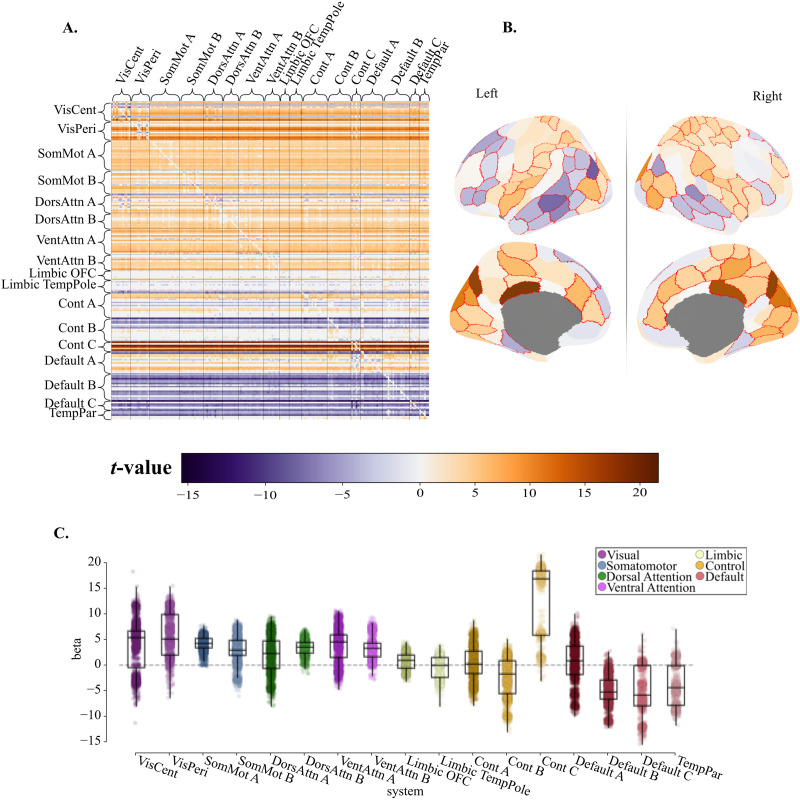
Nodal term group results. (A) Nodes that significantly related to awkwardness when controlling for each other node; FDR-corrected (*q* < 0.05). (B) Surface projections of the average beta weight for each node, as node weights were largely invariant across the horizontal axis in Panel A, unthresholded. Red border denotes significant (*q* < 0.05 FDR-corrected) nodal associations with awkwardness. (C) Boxplots decomposing the nodal term matrix by network; colors indicate 7-network labels.

We observed nodes that had significant activity associated with awkwardness ratings during movie-watching (Figure 3) after multiple comparisons correction (*q* < 0.05 FDR). Nodes whose activity was positively related to awkwardness included regions typically associated with the visual (e.g., bilateral visual cortex), somatomotor (e.g., primary sensory cortex, supplementary motor cortex), frontoparietal control (e.g., dorsal posterior cingulate cortex and bilateral insula), and default A (e.g., medial prefrontal, precuneus) subnetworks. Nodes whose activity was negatively related to awkwardness included regions typically associated with the default B, C, and temporoparietal networks (e.g., bilateral lateral middle temporal gyrus), which are often considered part of the broader default network (Andrews-Hanna et al., 2010), and left-lateralized nodes in ventrolateral and dorsolateral prefrontal cortices.

### Dynamic Functional Connectivity Associated With Awkwardness

**Figure 4.**
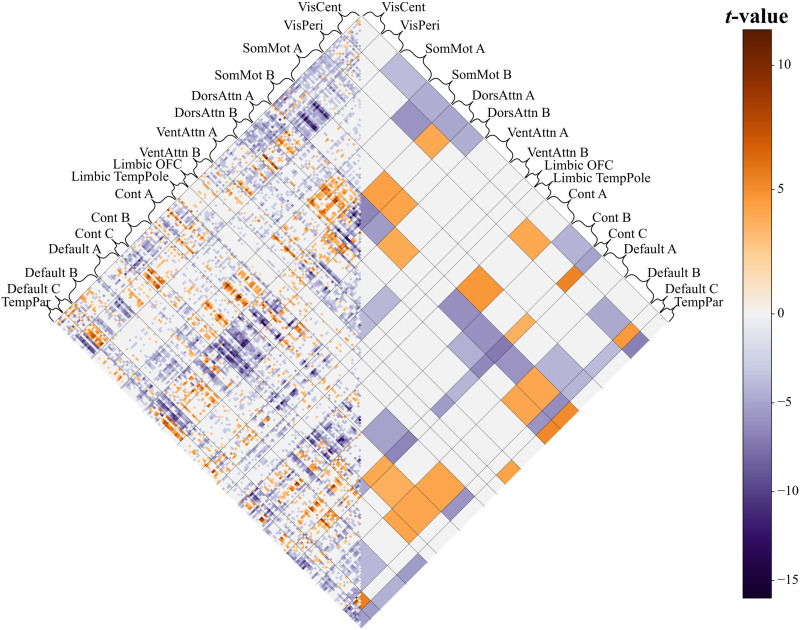
Connectivity term results. Left triangle: FDR-corrected (*q* < 0.05) suprathreshold edges; right triangle: significant network blocks assessed via spatial spin-test permutation, FDR-corrected (*q* < 0.05).

Next, we report edges (node-to-node) and networks (groups of edges) that had either significantly stronger or weaker connectivity associated with awkwardness ratings over time after multiple comparisons correction (Figure 4). For ease of interpretation, we describe network-level effects; that is, rather than focus on individual edges, we focus on pairs of networks within which significant edges are concentrated at a rate above what we expect from a null model. Moreover, we distinguish effects relating to connectivity within networks (e.g., edges between nodes within the default network) from effects relating to connectivity between networks (e.g., edges between nodes in the default and somatomotor networks). The distinction of within- versus between-network connectivity follows extensive prior work (e.g., Betzel et al., 2014; Hughes et al., 2020; Schurz et al., 2020) identifying these features as a core characteristic of the functional architecture of the brain (Sporns & Betzel, 2016).

Functional connectivity within networks (i.e., along the diagonal) was largely negatively associated with awkwardness. That is, the dorsal attention subnetwork A, frontoparietal control subnetwork C, and default subnetworks (B and temporoparietal) demonstrated decreased within-network connectivity as awkwardness increased. In contrast, functional connectivity within default subnetwork C—comprising parahippocampal and retrosplenial cortices and the inferior parietal lobule—was positively associated with awkwardness during movie-watching. Several studies suggest that networks defined using resting-state imaging data interact (i.e., have stronger functional connectivity) during externally oriented tasks and movie-watching (Betti et al., 2013; Betzel et al., 2020; Demirtaş et al., 2019; Finn & Bandettini, 2021; Gonzalez-Castillo & Bandettini, 2018). However, the specific complement of between-network effects is state- or behavior-dependent (Geerligs et al., 2015). Here, we observed numerous network interactions that positively and negatively related to awkwardness during movie-watching, which largely, but not exclusively, involved visual, dorsal attention, frontoparietal control, and default subnetworks. Statistical results for each within- and between-network effect are reported in Supporting Information Table S2.

### Control Analyses Using Visual Complexity Continuous Judgments

Due to our finding that visual network activity and connectivity were related to awkwardness, we conducted a sensitivity analysis relating activity and connectivity to awkwardness while controlling for subjective judgements of visual complexity using a continuous measure. This analysis allowed us to explore whether nonsocial factors (e.g., attention, low-level features of attention) contributed to the results, particularly in the visual cortex. To conduct this analysis, an independent cohort of young adults at Indiana University (*N* = 106; *M*_*Age*_ = 18.45 years, *SD* = 0.79 years) rated the same two videos on visual complexity in exchange for partial course credit. As with the awkwardness rating, the visual complexity rating was convolved with the Glover (Glover, 1999) hemodynamic response function via the “compute_regressor” function (Nilearn) (RRID:SCR_001362) to better represent the expected BOLD response. Visual complexity was unrelated to awkwardness (*r* = −0.066, *p* = 0.15) indicating that it was behaviorally distinct. Critically, we observed highly similar results for awkwardness when controlling for visual complexity. The correlation of the original model results and the visual complexity covariate model was significant across the activity term results (*r* = 0.99, *p* < 0.001) and the functional connectivity term results (*r* = 0.98, *p* < 0.001). This suggests that the observed patterns of activity and connectivity are related to awkwardness even when controlling for aspects of nonsocial visual processes (Supporting Information Figure S1).

### Quantifying Improvements in Model Fit

**Figure 5.**
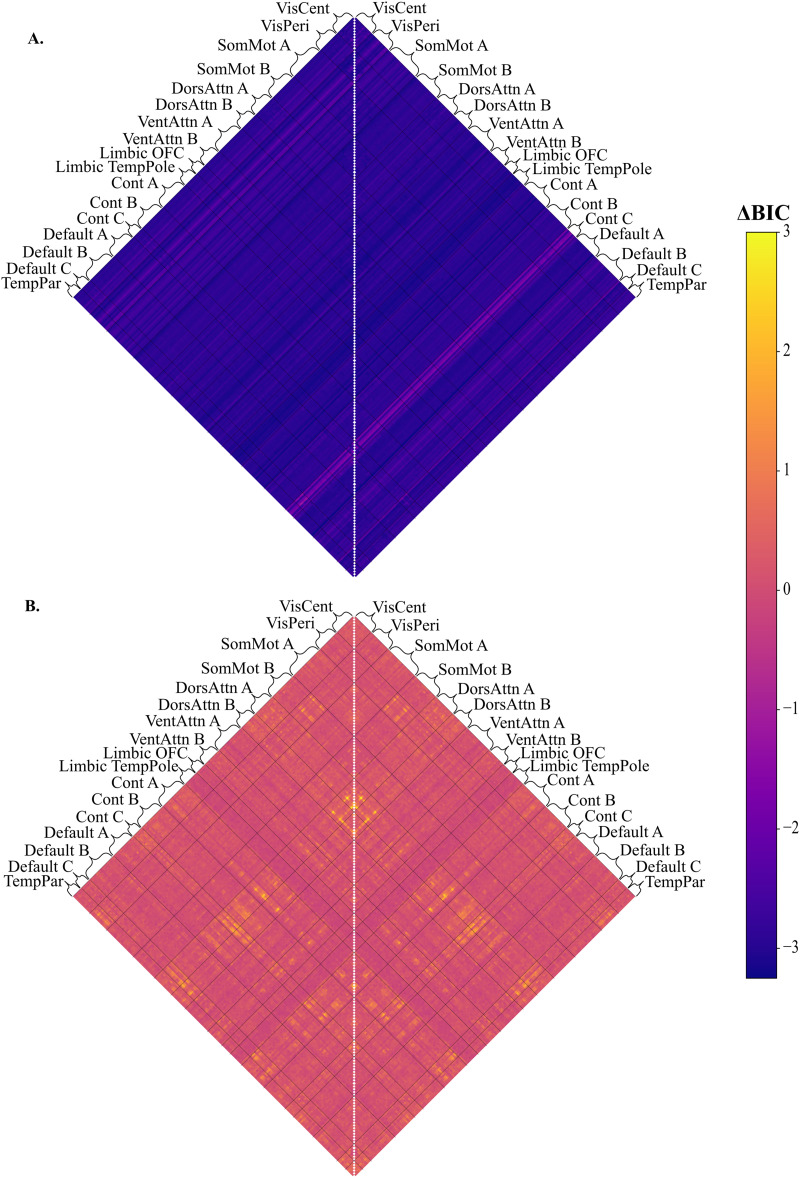
Average change (Δ) in model fit across stepwise inclusion of terms, left labels represent node *i*, while right labels represent node *j*, a positive change value indicates greater model improvement over previous model step. (A) Average change in Bayesian information criterion (BIC) across subjects between initial model containing only a single node and second step model containing a pair of nodes, displayed across all pairs of nodes. (B) Average change in BIC across subjects between secondary step model containing only a pair of nodes to the full model that includes the edge time series.

As an additional sensitivity analysis, we next sought to clarify the explicit improvements in model fit from each key term using a hierarchical framework. The benefit of this approach over the above analyses, which included terms for regional activity and functional connectivity in the same model, was that it could elucidate whether edge co-fluctuations added significantly increased variance explained with the inclusion of an additional node’s activity and then their edge co-fluctuation. Following the same modeling procedure as described previously, for each subject, we first fit a baseline model following the formula: *y* = *β_i_z_i_* + *β*_0_ + *ϵ*. Where *y* is the consensus awkwardness rating and *z*_*i*_ the standardized activity time series for node *i*. The second step included a second pair node: *j* into the model: *y* = *β_i_z_i_* + *β_j_z_j_* + *β*_0_ + *ϵ*. The final step was identical to the full model presented earlier with the inclusion of the edge time series as the interaction term between node *i* and node *j: y* = *β_i_z_i_* + *β_j_z_j_* + *β_ij_z_i_z_j_* + *β*_0_ + *ϵ*. Though some models benefitted from the inclusion of the second node, largely this was negligible. Across all subjects, 9.5% of the models demonstrated a significant improvement with the inclusion of a second node term (FDR-corrected for *q* < 0.05) while the inclusion of the edge time series term resulted in 90.1% of the models across all subjects, demonstrating a significant increase of model fit (Figure 5).

## DISCUSSION

The current study examined the unique contributions of both dynamic regional brain activity and functional connectivity within a single framework using a naturalistic measure of a theory of mind-related construct (awkwardness) alongside a novel analysis approach. We found that both dynamic functional connectivity and individual regional activations were separately and uniquely related to awkwardness. An important aspect of the current study is the use of a consensus awkwardness rating of the mockumentary-style show, *Nathan For You*, as a continuous proxy of awkwardness detection. This approach is similar to prior work examining regional activity associated with awkwardness detection in *The Office* (Pantelis et al., 2015). Critically, utilizing this proxy measure and building on prior work that simultaneously assesses dynamic activity and connectivity associated with behavior (Merritt et al., 2025), our contribution involves the mapping of complex social behavior to dynamic activations and connectivity, revealing the interactions between neural regions associated with naturalistic social cognitive processing.

Our finding that activity within unimodal networks, including visual and somatomotor networks, was positively associated with awkwardness is consistent with prior work that has implicated unimodal networks in naturalistic movie-watching (Wei et al., 2024). This suggests that unimodal networks may operate more independently—that is, with less correlated activity with other networks—than association networks. We note that while significant associations in these networks also emerged in the connectivity results, these were not within-network and instead reflected specific network-network interactions. Of particular note are the differences between central and peripheral networks. Both visual networks demonstrated strong positive regional activity with awkwardness; however, the two networks diverge in their functional connectivity relationships associated with awkwardness. The central visual network demonstrated negative functional connectivity relationships between networks, suggesting that while the activity of the visual network increased with awkwardness, it became more segregated from other networks as awkwardness increased. Conversely, the peripheral visual network demonstrated increased between-network integration as awkwardness increased, alongside an increase in regional activity of the peripheral visual network. Together, these findings may suggest that some regional activity associations are localized more independently, while connectivity associations were more prominent between networks. Put another way, the inclusion of edges within the model might have explained more variance in awkwardness because they captured how regions work together during movie-watching, though future work should explore this possibility further.

Alternatively, in the context of the movie stimuli used, rather than more traditional regional brain activity associated with theory of mind more broadly, awkwardness processing may instead have engaged cognitive mechanisms more akin to empathy, which is a related, but distinct, process from theory of mind (Dvash & Shamay-Tsoory, 2014; Kanske et al., 2017; Maliske, Schurz, & Kanske, 2023; Preckel et al., 2018). Indeed, work assessing the confluence of cognitive empathy and default networks demonstrates similar negative associations (Vemuri & Surampudi, 2015). Consistent with this assertion, we also found a positive association between nodal activation in the frontoparietal control network and the awkwardness ratings. The frontoparietal control C network is composed of the precuneus and posterior cingulate, which have been associated with affective theory of mind and empathy-related tasks (Carrington & Bailey, 2009; Marsh, 2018; Schurz et al., 2021; Timmers et al., 2018). Similar assessments of naturalistic social cognition, such as vicarious embarrassment, have shown regional activity in the insula, precuneus, and medial prefrontal cortex (Müller-Pinzler et al., 2015), as well as primary sensory cortices (Paulus et al., 2015). Consistent with these findings, we found that the strong nodal activity in the frontoparietal control C network and the between-network connectivity between visual peripheral and somatomotor B networks might reflect selective integration in these networks during awkwardness processing. Specifically, this may suggest that during naturalistic viewing, regions associated in previous work with vicarious embarrassment increase in activity associated with awkwardness, but additionally selectively integrate with sensory networks during periods of awkwardness. Vicarious embarrassment can be experienced in situations where social norms are violated even when one’s own social identity does not feel threatened (Kilian et al., 2018; Mayer et al., 2021; Miller, 1987; Ziegler et al., 2022), and has been linked behaviorally and neurally to measures of empathy (Krach et al., 2011; Melchers et al., 2015; Miller, 1987). This distributed activity between both sensorimotor networks alongside specific association networks such as the frontoparietal control C networks may indicate the complex engagement of these systems during the processing of awkwardness that relates to a distributed, but related, mechanistic process.

Beyond the sensorimotor associations present in the nodal results, we observed a significant negative relationship of activity and awkwardness within the default network (e.g., awkwardness increased, nodal activity in the default network decreased). At first glance, this may be surprising given that prior work found that continuous awkwardness processing was associated with heighted activation in regions within the default network (Pantelis et al., 2015). However, it is important to note that we did find evidence of default association with awkwardness in the connectivity results, specifically within default subnetwork C connectivity. The default C network, which is comprised of the retrosplenial and parahippocampal cortices, has been implicated in both theory of mind and autobiographical memory (Hughes et al., 2024; Spreng et al., 2016), potentially indicating this process may be related to autobiographical or self-referential processes. Future work should examine the relationship between dynamic connectivity in the default mode with dynamic behavior, rather than pure assessment of static associations.

More broadly, the functional connectivity findings of this work that relate to awkwardness generally occurred between networks. This is unsurprising given the demonstration of stronger network interactions during task performance (Bola & Sabel, 2015; Moraschi et al., 2020), including theory of mind tasks (Maliske, Lehmann, et al., 2023; Maliske, Schurz, & Kanske, 2023). Specifically, this work suggests that increased functional connectivity between networks is related to the complexity of the stimuli processing, with greater complexity requiring more internetwork interactions (Di & Biswal, 2020; Li et al., 2020; Simony et al., 2016). This finding indicates a level of functional reorganization dependent on the awkwardness rating; the reorganization is particularly apparent when comparing within- and between-network associations. Specifically, the default C network demonstrates increased within-network connectivity and between-network connectivity with the visual peripheral network associated with awkwardness. The increased integration of these networks associated with awkwardness may indicate there is context-dependent visual information related to awkwardness that is associated with downstream sociocognitive processes. Indeed, prior work has found an increased functional connectivity of the retrosplenial cortex and the visual cortex in association with social thoughts of the future (Villena-Gonzalez et al., 2018). Conversely, we also found that between-network connectivity between the default C network and the somatomotor, dorsal attention, and frontoparietal control A networks were negatively associated with awkwardness. One possible interpretation for this finding is that there was a suppression between these networks as other systems positively associated with awkwardness came “online.” Supporting this assertion, the dorsal attention network is often anticorrelated with the default network (Esposito et al., 2018; Gopinath et al., 2015; Uddin et al., 2009), though work has shown this to be context dependent (Dixon et al., 2017). In the context of the current study, these findings could account for the increased between-network connectivity (e.g., between the dorsal attention, default B, and temporoparietal networks) but decreased within-network connectivity (e.g., the default B and temporoparietal networks) in response to awkwardness. Simply put, these findings may indicate a level of contextual and spatially dependent integration with the default networks.

An important finding in the current work is that, based on our hierarchical framework, the inclusion of dynamic functional connectivity to the model significantly increased variance explained across ~90% of edges across all subjects. This finding critically extends prior work showing that dynamic changes in functional connectivity may have greater sensitivity in detection of cognitive and clinical differences than static measures of functional connectivity (Chen et al., 2017; Chumin et al., 2024; Cutts et al., 2025; Damaraju et al., 2014), though it is important to note we did not directly compare these results to static connectivity. However, our finding suggests there is unique variance explained associated with dynamic functional connectivity that cannot be explained by regional activity alone. It is important to note that dynamic nodal activity also significantly and uniquely predicted behavior across several notable sets of regions (visual, somatomotor, and frontoparietal control network C, among others). Thus, both regional and functional connectivity uniquely contribute to social cognition.

The method used in the current study is not the first to attempt to model concurrent regional activity and functional connectivity in relation to behavior. Indeed, psychophysiological interaction (PPI) is a common method that aims to model similar relationships. However, an important benefit of our approach is that it is nondirectional. That is, PPI analyses are used most often to assess only specific “seed” regions, often previously defined by the task related activity, though see Gerchen et al. (2014) and Ni et al. (2024). Thus, the resulting functional connectivity estimates are directional, that is, nonsymmetric. Such findings are more difficult to interpret and validate (Mill et al., 2017). An advantage of the method we utilize is that it results in more interpretable results, akin to more common representations of whole-brain functional connectivity. Another difference between these methods is that one behavior serves as the outcome of interest under the current approach, unlike PPI wherein multiple behaviors can serve as predictors. While the current approach could be extended to event-related designs for more direct comparison, it is unclear if the discontinuity of behavioral responses under those paradigms would be better served by PPI or the current method. Ultimately, future work should clarify the best method depending on study goal and design.

Some limitations in this approach should be noted. Despite demonstrating significant associations of awkwardness to neural activity and connectivity that are robust to additional variables, the average variance explained within a single univariate model was relatively low. This may in part be due to a reliance on an independent consensus awkwardness rating, rather than leveraging participant-specific perceptions of awkwardness. It is important to note that there was high reliability among the consensus group about which moments were awkward, suggesting the suitability of the independent consensus approach. However, future work—when performed using data from more heterogenous groups, for example, older adult or clinical populations—should consider the individual variability of the ratings to better model individual variance. In addition, our robust prewhitening procedure, though effective in reducing spurious correlations driven by serial correlations (Parlak et al., 2023), nonetheless resulted in reductions in variance explained by the model. Indeed, performing an identical analysis stream without the prewhitening step resulted in increased (more than double) variance explained (see Supporting Information Figure S2). It is also worth noting that the pattern of results was highly similar across both analysis streams. Because model fit is rarely reported in fMRI mass univariate analyses (Monti, 2011; Razavi et al., 2003), or in similar approaches with edge time series (Jones et al., 2024), it is difficult to assess the relative standing of our model fit in comparison. Disentangling awkwardness from other related features to gain specificity about social cognitive mechanisms could be served by simultaneous physiological measurements.

Our sensitivity analysis addressed one such potential confound: low-level visual properties of the scenes in our paradigm. The fact that results remained unchanged when including this control suggests that the reported effects, particularly within visual systems, were not driven by broad visual scene properties. However, we cannot rule out the possibility that other confounds, including interoceptive awareness, contributed to these findings. Indeed, prior work has shown that heart beat counting related to naturalistic deception detection, or another form of social cognition (Heemskerk et al., 2024) have neural correlates that partially overlap with theory of mind (Hughes et al., 2025). Investigations seeking to distinguish social cognitive processes could therefore control for alternate features than in the current work.

An important and related limitation in the current study is that the results should be interpreted with caution in the context of awkwardness. A key challenge of using naturalistic stimuli to study social cognition is that perceptual, affective, and attentional processes are deeply intertwined during real-world social experiences (e.g., Redcay & Moraczewski, 2020). For example, low-level features, including luminance or loudness, could covary with awkwardness in naturalistic video stimuli, thus contributing to the pattern of results observed. Alternatively, emotional responses, such as arousal, could have driven these results. Indeed, prior work suggests that feelings of embarrassment, which are frequently elicited by socially awkward situations (e.g., Finger et al., 2006), are closely intertwined with arousal (Singh & Bhushan, 2025). Though our sensitivity analyses accounted for one low-level feature, visual complexity, we did not consider other possible confounds because doing so would have risked removing variance that was central to the construct of awkwardness. While such an approach would more directly connect our neural results to awkwardness specifically, this was beyond the scope of the current investigation. Future work should systematically identify core underlying components of interest with awkwardness (e.g., loudness, luminance, arousal) and demonstrate to what degree that they may be contextually associated with the stimuli while remaining conceptually unrelated to awkwardness itself.

Taken together, both regional brain activity and functional connectivity are separably associated with the aspect of social cognition we assessed, awkwardness. As current work often only assesses one or the other of regional activity or functional connectivity, we demonstrate that this practice may be under- or overcompensating the associations between social cognition and any single one of these measures. As social cognition is complex, composed of myriad features beyond just the awkwardness measure assessed here, greater understanding of the combinations of regional brain activity and functional connectivity as they apply to these features may provide insight into the underlying mechanisms that make up social cognition.

## ACKNOWLEDGMENTS

This work was supported by R01AG075044 (PIs: Krendl, Betzel) and R01AG070931 (PI: Krendl) from the National Institute on Aging. The content is solely the responsibility of the authors and does not necessarily represent the official views of the National Institutes of Health. The authors thank Samuel Rincón, Lucas Hamilton, Amy Gourley, Skylar Wilson, and Carter Wittendorf.

## SUPPORTING INFORMATION

Supporting information for this article is available at https://doi.org/10.1162/NETN.a.571.

## AUTHOR CONTRIBUTIONS

Roberto C. French: Conceptualization; Data curation; Formal analysis; Investigation; Methodology; Software; Visualization; Writing – original draft. Haily Merritt: Conceptualization; Formal analysis; Investigation; Methodology; Resources; Supervision; Validation; Writing – review & editing. Colleen Hughes: Conceptualization; Data curation; Investigation; Methodology; Resources; Supervision; Validation; Writing – review & editing. Richard Betzel: Conceptualization; Funding acquisition; Investigation; Methodology; Project administration; Resources; Supervision; Validation; Writing – review & editing. Anne C. Krendl: Conceptualization; Funding acquisition; Investigation; Methodology; Project administration; Resources; Supervision; Validation; Writing – review & editing.

## FUNDING INFORMATION

Anne Krendl, National Institute on Aging (https://dx.doi.org/10.13039/100000049), Award ID: R01AG075044. Anne Krendl, National Institute on Aging (https://dx.doi.org/10.13039/100000049), Award ID: R01AG070931.

## Supplementary Material



## TECHNICAL TERMS

Functional connectivity: A statistical measurement of the similarity of two brain regions’ neural signal. In fMRI, most often captured as the product-moment correlation.
Dynamic functional connectivity: A measurement of functional connectivity broken down into discrete time points or windows, allowing for the assessment of change in functional connectivity across time.
BOLD signal: Blood-oxygen-level-dependent signal; a signal measured during functional magnetic resonance imaging capturing the level of blood oxygenation across time, which acts as a proxy for neural activity.
Edge time series: A particular application of dynamic functional connectivity, conceptually assessing the momentary functional coupling between two brain regions at single time points.
Ecological validity: A term that explains the level to which an experimental design mirrors the real-world application of what the experiment is assessing.
Theory of mind: One’s ability to infer the thoughts, motivations, and emotions of another.
PPI: Psychophysiological interaction; an analytical framework that modifies the traditional general linear model used in event related fMRI analyses to include the BOLD signal from another brain region in the analysis. This signal is included both as an independent term, and in an interaction with the event-related task regressors.
