# Supplementary material for: Regional activity and interregional functional connectivity uniquely contribute to social cognitive judgments during movie-watching
Source: Netw Neurosci. 2026 Jul 27;10(3):738–60. doi: 10.1162/NETN.a.571 (PMC13418520; doi:10.1162/NETN.a.571)
Supplement: Supplementary file 1 [file netn-10-3-738-s001.pdf]

Supplementary materials

Detailed image preprocessing

A *B0*-nonuniformity map (fieldmap) was estimated based on two echo-planar imaging (EPI) references with topup (Smith et al., 2004) (FSL 6.0.5.1:57b01774).

Anatomical data preprocessing. The T1w anatomical image was corrected for intensity non-uniformity with ‘N4BiasFieldCorrection’ (Tustison et al., 2010) distributed with ANTs 2.5.0 (Avants et al., 2008) (RRID: SCR_004757) and used as T1w-reference throughout the workflow. The T1w-reference was then skull-stripped with a Nipype implementation of the ‘antsBrainExtraction.sh’ workflow (from ANTs), using NKI as target template. Brain tissue segmentation of cerebrospinal fluid (CSF), white-matter (WM) and gray-matter (GM) was performed on the brain-extracted T1w using ‘fast’ (Zhang et al., 2001) (FSL; RRID: SCR_002823). Brain surfaces were reconstructed using ‘recon-all’ (Dale et al., 1999) (FreeSurfer 7.3.2, RRID: SCR_001847), and the brain mask estimated previously was refined with a custom variation of the method to reconcile ANTs-derived and FreeSurfer-derived segmentations of the cortical gray-matter of Mindboggle (Klein et al., 2017) (RRID: SCR_002438). Volume-based spatial normalization to MNI152NLin6Asym (Evans et al., 2012), accessed with TemplateFlow (23.1.0) (Ciric et al., 2022), was performed through nonlinear registration with ‘antsRegistration’ (ANTs 2.5.0), using brain-extracted versions of both T1w reference and the T1w template.

Functional data preprocessing. For functional images, a reference volume was generated, using a custom methodology of fMRIPrep, for use in head motion correction. Head-motion parameters with respect to the BOLD reference (transformation matrices, and six corresponding rotation and translation parameters) are estimated before any spatiotemporal filtering using ‘mcflirt’ (FSL) (Jenkinson et al., 2002). The estimated fieldmap was then aligned

with rigid-registration to the target EPI reference run. The field coefficients were mapped on to the reference EPI using the transform. The BOLD reference was then co-registered to the T1w reference using 'bbregister' (FreeSurfer) which implements boundary-based registration (Greve & Fischl, 2009). Co-registration was configured with six degrees of freedom. All resamplings were performed with a single interpolation step by composing all the pertinent transformations (i.e. head-motion transform matrices, susceptibility distortion correction, and co-registrations to anatomical and template spaces). Gridded (volumetric) resamplings were performed using 'nitransforms', configured with cubic B-spline interpolation.

For subsequent denoising, several confounding time-series were calculated based on the preprocessed BOLD images co-registered to the T1w reference: three region-wise global signals extracted within the CSF, the WM, and the global masks. The head-motion estimates calculated in the correction step and discrete cosine regressors (analogous to a high-pass filter at 0.008 hertz) were also placed within the corresponding confounds file. The confound time series derived from head motion estimates and global signals were expanded with the inclusion of temporal derivatives and quadratic terms for each (Satterthwaite et al., 2013).

For functional images, a reference volume was generated, using a custom methodology of fMRIPrep, for use in head motion correction. Head-motion parameters with respect to the BOLD reference (transformation matrices, and six corresponding rotation and translation parameters) are estimated before any spatiotemporal filtering using 'mcflirt' (FSL) (Jenkinson et al., 2002). The estimated fieldmap was then aligned with rigid-registration to the target EPI reference run. The field coefficients were mapped on to the reference EPI using the transform. The BOLD reference was then co-registered to the T1w reference using 'bbregister' (FreeSurfer) which implements boundary-based registration (Greve & Fischl, 2009). Co-registration was configured

with six degrees of freedom. All resamplings were performed with a single interpolation step by composing all the pertinent transformations (i.e. head-motion transform matrices, susceptibility distortion correction, and co-registrations to anatomical and template spaces). Gridded (volumetric) resamplings were performed using 'nitransforms', configured with cubic B-spline interpolation.

For subsequent denoising, several confounding time-series were calculated based on the preprocessed BOLD images co-registered to the T1w reference: three region-wise global signals extracted within the CSF, the WM, and the global masks. The head-motion estimates calculated in the correction step and discrete cosine regressors (analogous to a high-pass filter at 0.008 hertz) were also placed within the corresponding confounds file. The confound time series derived from head motion estimates and global signals were expanded with the inclusion of temporal derivatives and quadratic terms for each (Satterthwaite et al., 2013).

Denoising. Subsequent denoising appropriate for functional connectivity analyses was conducted using the eXtensible Connectivity Pipeline- DCAN (XCP-D) (Ciric et al., 2018; Satterthwaite et al., 2013). The first two volumes were discarded from each run as non-steady state volumes. High-motion outlier time points were identified for later censoring using framewise displacement (FD) greater than 0.5mm. Nuisance regressors included the fMRIPrep-calculated confounding timeseries for the three region-wise global signals extracted within the cerebrospinal fluid, white matter, and global masks, six head motion parameters, the temporal derivatives and quadratic terms for those nine terms, and discrete cosine regressors (analogous to a high-pass filter at 0.008 Hz), and linear trend and intercept terms. After denoising, the 200-region Schaefer parcellation (Schaefer et al., 2018) was applied to the residual BOLD signal. Parcellated timeseries for the two movie scans were then concatenated.

Supplementary Table 1.

Region (parcel)	Nodal axis mean	R	A	S
LH_VisCent_ExStr_1	5.57329	-26	-77	-14
LH_VisCent_ExStr_2	-0.254207	-27	-95	-12
LH_VisCent_Striate_1	6.95455	-5	-93	-4
LH_VisCent_ExStr_3	-0.279313	-23	-97	6
LH_VisCent_ExStr_4	4.83809	-40	-85	11
LH_VisCent_ExStr_5	5.20212	-23	-87	23
LH_VisPeri_ExStrInf_1	-0.00533949	-24	-53	-9
LH_VisPeri_ExStrInf_2	5.48973	-10	-67	-4
LH_VisPeri_ExStrInf_3	-0.0391204	-14	-44	-3
LH_VisPeri_StriCal_1	4.37485	-11	-70	7
LH_VisPeri_ExStrSup_1	8.69279	-12	-73	22
LH_VisPeri_ExStrSup_2	10.6605	-7	-87	28
LH_SomMotA_1	0.106978	-7	-12	46
LH_SomMotA_2	0.051511	-49	-28	57
LH_SomMotA_3	3.62636	-40	-25	57
LH_SomMotA_4	0.483373	-32	-22	64
LH_SomMotA_5	4.60407	-26	-38	68
LH_SomMotA_6	0	-20	-11	68
\LH_SomMotA_7	4.62783	-5	-29	67
LH_SomMotA_8	4.84998	-19	-31	68
LH_SomMotB_Aud_1	0.22913	-51	-4	-2
LH_SomMotB_Aud_2	-0.0200349	-53	-24	9
LH_SomMotB_S2_1	5.67486	-37	-21	16
LH_SomMotB_S2_2	1.91169	-55	-4	10
LH_SomMotB_Aud_3	0	-56	-40	20
LH_SomMotB_S2_3	2.74273	-53	-22	18
LH_SomMotB_Cent_1	0	-56	-8	31
LH_SomMotB_Cent_2	0.0899534	-47	-9	46
LH_DorsAttnA_TempOcc_1	-3.12878	-43	-48	-19
LH_DorsAttnA_TempOcc_2	2.1359	-45	-69	-8
LH_DorsAttnA_ParOcc_1	7.62314	-47	-70	10
LH_DorsAttnA_SPL_1	0	-26	-70	38
LH_DorsAttnA_SPL_2	-0.0945769	-17	-73	54
LH_DorsAttnA_SPL_3	1.3229	-29	-60	59
LH_DorsAttnB_PostC_1	3.42587	-54	-27	42
LH_DorsAttnB_PostC_2	0.255954	-41	-35	47
LH_DorsAttnB_PostC_3	0.0172926	-31	-46	63
LH_DorsAttnB_PostC_4	0	-17	-53	68

LH_DorsAttnB_FEF_1	0	-31	-4	53
LH_SalVentAttnA_ParOper_1	2.62274	-61	-26	28
LH_SalVentAttnA_Ins_1	6.27705	-39	-4	-4
LH_SalVentAttnA_FrOper_1	4.15815	-39	1	11
LH_SalVentAttnA_FrOper_2	0	-51	9	11
LH_SalVentAttnA_ParMed_1	6.78418	-11	-35	46
LH_SalVentAttnA_FrMed_1	0.241378	-6	9	41
LH_SalVentAttnA_FrMed_2	0	-6	-3	65
LH_SalVentAttnB_IPL_1	0	-60	-39	36
LH_SalVentAttnB_PFCl_1	0	-28	43	31
LH_SalVentAttnB_Ins_1	0.25075	-33	20	5
LH_SalVentAttnB_PFCmp_1	6.07454	-6	30	25
LH_LimbicB_OFC_1	0	-24	22	-20
LH_LimbicB_OFC_2	0	-10	35	-21
LH_LimbicA_TempPole_1	0.0532888	-29	-6	-39
LH_LimbicA_TempPole_2	-0.0370172	-45	-20	-30
LH_LimbicA_TempPole_3	0	-28	10	-34
LH_LimbicA_TempPole_4	0	-43	8	-19
LH_ContA_Temp_1	-5.25083	-57	-60	-1
LH_ContA_IPS_1	0.597459	-35	-62	48
LH_ContA_IPS_2	3.2875	-45	-42	46
LH_ContA_IPS_3	5.23472	-33	-49	47
LH_ContA_PFCd_1	-0.0169428	-22	6	62
LH_ContA_PFClv_1	0	-42	40	16
LH_ContA_PFCl_1	-2.17356	-44	20	27
LH_ContA_PFCl_2	0	-48	6	29
LH_ContA_PFCl_3	-0.349987	-43	6	43
LH_ContA_Cingm_1	0	-3	4	30
LH_ContB_Temp_1	-8.36626	-61	-43	-13
LH_ContB_IPL_1	-1.99267	-53	-51	46
LH_ContB_PFCl_1	-6.0378	-40	19	49
LH_ContB_PFClv_1	-5.94383	-42	49	-6
LH_ContB_PFClv_2	0.0170953	-28	58	8
LH_ContC_pCun_1	14.7731	-9	-73	38
LH_ContC_pCun_2	0.0216768	-6	-60	57
LH_ContC_Cingp_1	16.1417	-5	-29	28
LH_DefaultA_IPL_1	-5.50477	-46	-66	38
LH_DefaultA_PFCd_1	-7.9505	-24	25	49
LH_DefaultA_pCunPCC_1	0.291958	-5	-55	27
LH_DefaultA_pCunPCC_2	0.284975	-4	-31	36
LH_DefaultA_pCunPCC_3	0.162248	-6	-54	42

LH_DefaultA_PFCm_1	-0.416641	-6	36	-10
LH_DefaultA_PFCm_2	0	-12	63	-6
LH_DefaultA_PFCm_3	1.0983	-6	44	7
LH_DefaultB_Temp_1	-4.00911	-47	8	-33
LH_DefaultB_Temp_2	-5.88866	-60	-19	-22
LH_DefaultB_Temp_3	-6.38155	-56	-6	-12
LH_DefaultB_Temp_4	-10.0275	-58	-30	-4
LH_DefaultB_IPL_1	-4.74836	-57	-54	28
LH_DefaultB_PFCd_1	-4.58085	-8	59	21
LH_DefaultB_PFCd_2	-7.85488	-11	47	45
LH_DefaultB_PFCd_3	-0.228428	-3	33	43
LH_DefaultB_PFCd_4	-5.50805	-9	17	63
LH_DefaultB_PFCv_1	0	-35	20	-13
LH_DefaultB_PFCv_2	0	-32	42	-13
LH_DefaultB_PFCv_3	-7.07096	-46	31	-7
LH_DefaultB_PFCv_4	-6.19034	-52	22	8
LH_DefaultC_IPL_1	-11.0445	-39	-80	31
LH_DefaultC_Rsp_1	-0.106001	-11	-56	13
LH_DefaultC_PHC_1	-6.27222	-26	-32	-18
LH_TempPar_1	-8.95806	-58	-43	7
LH_TempPar_2	-3.95563	-48	-57	18
RH_VisCent_ExStr_1	6.76818	29	-69	-12
RH_VisCent_ExStr_2	7.55143	48	-71	-6
RH_VisCent_Striate_1	6.0669	11	-92	-5
RH_VisCent_ExStr_3	-0.281871	31	-94	-4
RH_VisCent_ExStr_4	6.8878	42	-80	10
RH_VisCent_ExStr_5	10.0836	20	-90	22
RH_VisPeri_ExStrInf_1	7.50847	12	-65	-5
RH_VisPeri_ExStrInf_2	-0.0684102	16	-46	-1
RH_VisPeri_StriCal_1	6.36585	9	-75	9
RH_VisPeri_ExStrSup_1	2.67185	22	-60	7
RH_VisPeri_ExStrSup_2	10.7057	11	-74	26
RH_VisPeri_ExStrSup_3	8.70615	16	-85	39
RH_SomMotA_1	0	51	-22	52
RH_SomMotA_2	3.70221	47	-11	48
RH_SomMotA_3	0.0366437	7	-11	51
RH_SomMotA_4	2.38545	40	-24	57
RH_SomMotA_5	5.85045	32	-40	64
RH_SomMotA_6	4.36004	33	-21	65
RH_SomMotA_7	5.24609	29	-34	65
RH_SomMotA_8	0.709964	22	-9	67

RH_SomMotA_9	4.63152	10	-39	69
RH_SomMotA_10	2.83475	6	-23	69
RH_SomMotA_11	3.42475	20	-29	70
RH_SomMotB_Aud_1	0.106574	51	-15	5
RH_SomMotB_Aud_2	-2.38002	64	-23	8
RH_SomMotB_S2_1	4.47665	38	-13	15
RH_SomMotB_S2_2	6.63588	44	-27	18
RH_SomMotB_S2_3	0.0341947	59	0	10
RH_SomMotB_S2_4	0	56	-11	14
RH_SomMotB_Cent_1	0	58	-5	31
RH_DorsAttnA_TempOcc_1	-0.0257393	50	-53	-15
RH_DorsAttnA_ParOcc_1	5.47904	52	-60	9
RH_DorsAttnA_SPL_1	-0.0163358	33	-75	32
RH_DorsAttnA_SPL_2	0.104673	15	-73	53
RH_DorsAttnA_SPL_3	5.43557	34	-48	51
RH_DorsAttnA_SPL_4	2.7271	26	-61	58
RH_DorsAttnB_PostC_1	0	59	-16	34
RH_DorsAttnB_PostC_2	1.93843	41	-31	46
RH_DorsAttnB_PostC_3	3.24917	8	-56	61
RH_DorsAttnB_PostC_4	0.216834	21	-48	70
RH_DorsAttnB_FEF_1	5.28135	34	-4	52
RH_SalVentAttnA_ParOper_1	5.64058	60	-26	27
RH_SalVentAttnA_PrC_1	0	51	4	40
RH_SalVentAttnA_Ins_1	4.0872	41	6	-15
RH_SalVentAttnA_Ins_2	3.16223	46	-4	-4
RH_SalVentAttnA_FrOper_1	3.20547	43	7	4
RH_SalVentAttnA_FrMed_1	3.93277	7	9	41
RH_SalVentAttnA_ParMed_1	5.24994	10	-15	41
RH_SalVentAttnA_ParMed_2	8.79606	11	-36	47
RH_SalVentAttnA_FrMed_2	-0.0531516	8	3	66
RH_SalVentAttnB_IPL_1	0	62	-37	37
RH_SalVentAttnB_PFClv_1	0.284793	43	45	10
RH_SalVentAttnB_PFCl_1	4.12259	30	48	27
RH_SalVentAttnB_Ins_1	0.334469	34	21	-8
RH_SalVentAttnB_Ins_2	0.188257	36	24	5
RH_SalVentAttnB_PFCmp_1	5.77657	7	31	28
RH_LimbicB_OFC_1	0	12	39	-22
RH_LimbicB_OFC_2	0	28	22	-19
RH_LimbicB_OFC_3	0	5	37	-14
RH_LimbicB_OFC_4	0.841639	15	64	-8
RH_LimbicA_TempPole_1	0.0181681	30	9	-38

RH_LimbicA_TempPole_2	0	47	-12	-35
RH_LimbicA_TempPole_3	0	25	-11	-32
RH_LimbicA_TempPole_4	-0.392101	39	-35	-23
RH_ContA_IPS_1	0.281105	37	-63	47
RH_ContA_IPS_2	2.05202	46	-38	49
RH_ContA_PFCd_1	0.0732077	26	7	58
RH_ContA_PFCl_1	0	52	11	21
RH_ContA_PFCl_2	0	46	24	26
RH_ContA_Cingm_1	0	5	3	30
RH_ContB_Temp_1	-1.98645	61	-13	-21
RH_ContB_Temp_2	-5.38231	63	-41	-12
RH_ContB_IPL_1	0.0199806	51	-59	44
RH_ContB_IPL_2	0	53	-42	48
RH_ContB_PFCld_1	0.0178852	41	33	37
RH_ContB_PFCld_2	0	42	14	49
RH_ContB_PFClv_1	0	36	46	-13
RH_ContB_PFClv_2	0	29	58	5
RH_ContB_PFCmp_1	0	7	25	55
RH_ContB_PFCld_3	-0.809352	23	24	53
RH_ContC_pCun_1	14.3318	14	-70	37
RH_ContC_pCun_2	5.19719	6	-58	44
RH_ContC_Cingp_1	14.0144	5	-24	31
RH_DefaultA_IPL_1	0	54	-50	28
RH_DefaultA_PFCd_1	-0.0170726	29	30	42
RH_DefaultA_pCunPCC_1	4.84925	7	-49	31
RH_DefaultA_PFCm_1	4.8885	8	42	4
RH_DefaultA_PFCm_2	0	6	29	15
RH_DefaultA_PFCm_3	0	8	58	18
RH_DefaultB_Temp_1	-5.57586	63	-27	-6
RH_DefaultB_AntTemp_1	0	47	13	-30
RH_DefaultB_PFCd_1	-4.17064	15	46	44
RH_DefaultB_PFCv_1	-0.54727	51	28	0
RH_DefaultC_IPL_1	-7.72857	47	-69	27
RH_DefaultC_Rsp_1	-0.121453	12	-55	15
RH_DefaultC_PHC_1	-1.56692	28	-36	-14
RH_TempPar_1	-5.00551	55	-6	-10
RH_TempPar_2	-8.37402	52	-31	2
RH_TempPar_3	0.00123446	57	-45	9
RH_TempPar_4	0.0689035	60	-39	17

Supplementary Table 1. Full nodal mean *t*-statistic values, averaged across invariant axis. RAS coordinates of the parcel centroid in MNI152 1mm space

Supplementary Table 2.

System names	Mean t-statistic
VisCent - SomMot A	-1.232791334
VisCent - SomMot B	-1.605123494
VisCent - DorsAttn A	-1.898749343
VisCent - DorsAttn B	-1.874863658
VisCent - Cont C	-2.151512899
VisCent - Default C	-0.050597195
VisPeri - SomMot B	-5.059007081
VisPeri - DorsAttn A	-0.041678104
VisPeri - DorsAttn B	-0.084351765
VisPeri - Cont A	0.072173456
VisPeri - Cont C	2.211913946
VisPeri - Default B	-1.674761901
VisPeri - Default C	2.48074501
VisPeri - TempPar	-3.757847665
SomMot B - DorsAttn A	0.93985997
SomMot B - DorsAttn B	1.38013118
SomMot B - Cont A	0.872421925
SomMot B - Default B	-1.085732553
DorsAttn A - DorsAttn A	-3.039461984
DorsAttn A - DorsAttn B	-1.741291431
DorsAttn A - VentAttn A	0.12242464
DorsAttn A - Cont A	-2.133293293
DorsAttn A - Cont B	-3.976579865
DorsAttn A - Cont C	-5.75718719
DorsAttn A - Default A	-1.959870261
DorsAttn A - Default B	-0.153455292
DorsAttn A - Default C	-4.672317436
DorsAttn A - TempPar	0.260148378
DorsAttn B - DorsAttn B	-1.435640743
DorsAttn B - Cont B	-2.423259695
DorsAttn B - Cont C	-6.196864165
DorsAttn B - Default B	0.58948615
DorsAttn B - Default C	-4.47869759
DorsAttn B - TempPar	2.119648384
VentAttn A - Cont A	-0.141345508
VentAttn A - Cont C	-1.942776867
VentAttn B - Cont C	-2.253561452
VentAttn B - Default C	-0.136683717
VentAttn B - TempPar	0.641814375
Cont A - Cont B	-2.429884269

Cont A - Cont C	-3.427687107
Cont A - Default A	-1.227843103
Cont A - Default B	-0.28637854
Cont A - Default C	-2.961451631
Cont A - TempPar	-0.165210009
Cont B - Cont C	-0.18712493
Cont B - Default B	0.301736439
Cont B - TempPar	0.004889308
Cont C - Cont C	-2.192644081
Cont C - Default A	0.395700312
Cont C - Default B	1.216012932
Cont C - Default C	0.082492902
Default A - TempPar	-2.305415643
Default B - Default B	-1.386904894
Default B - TempPar	-1.637919347
Default C - Default C	1.692507454
Default C - TempPar	-4.142217206
TempPar - TempPar	-3.087615887

Supplementary Table 2. Significant system blocks in group level connectivity results.

Supplementary Figure 1.

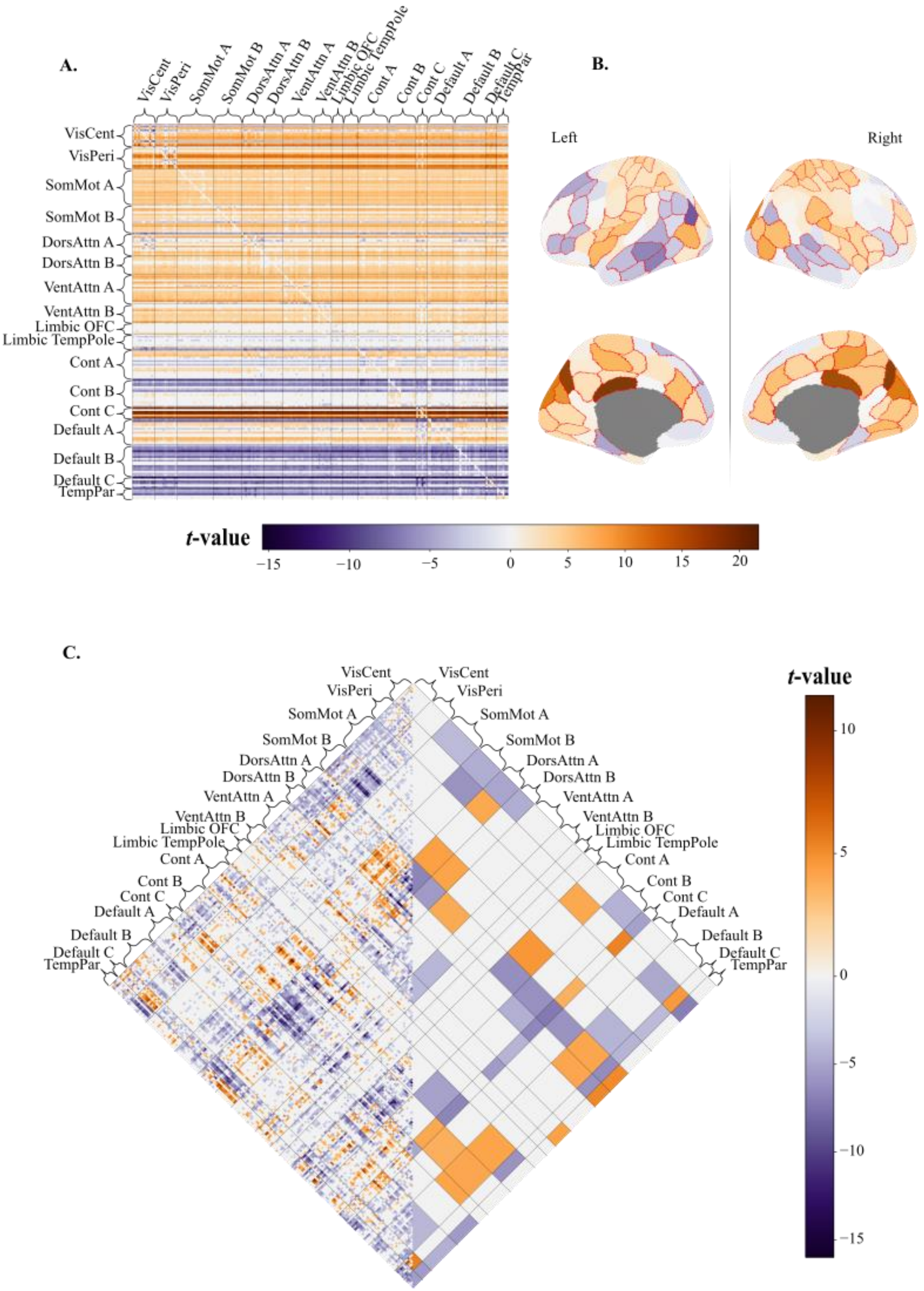

Supplementary figure 1. Group level results of model containing visual complexity consensus rating as confound. A. Nodal level suprathreshold results, FDR-corrected ($q < 0.05$), B. surface projection of nodal level results, averaged across horizontal axis of A, unthresholded. Red border denotes significant ($q < 0.05$ FDR corrected) nodal associations with awkwardness. C. Edge term results, left triangle displays FDR-corrected suprathreshold edges ($q < 0.05$), right triangle displays spatial spin-test permutation (FDR-corrected, $q < 0.05$) significant network blocks.

Supplementary Figure 2.

A.

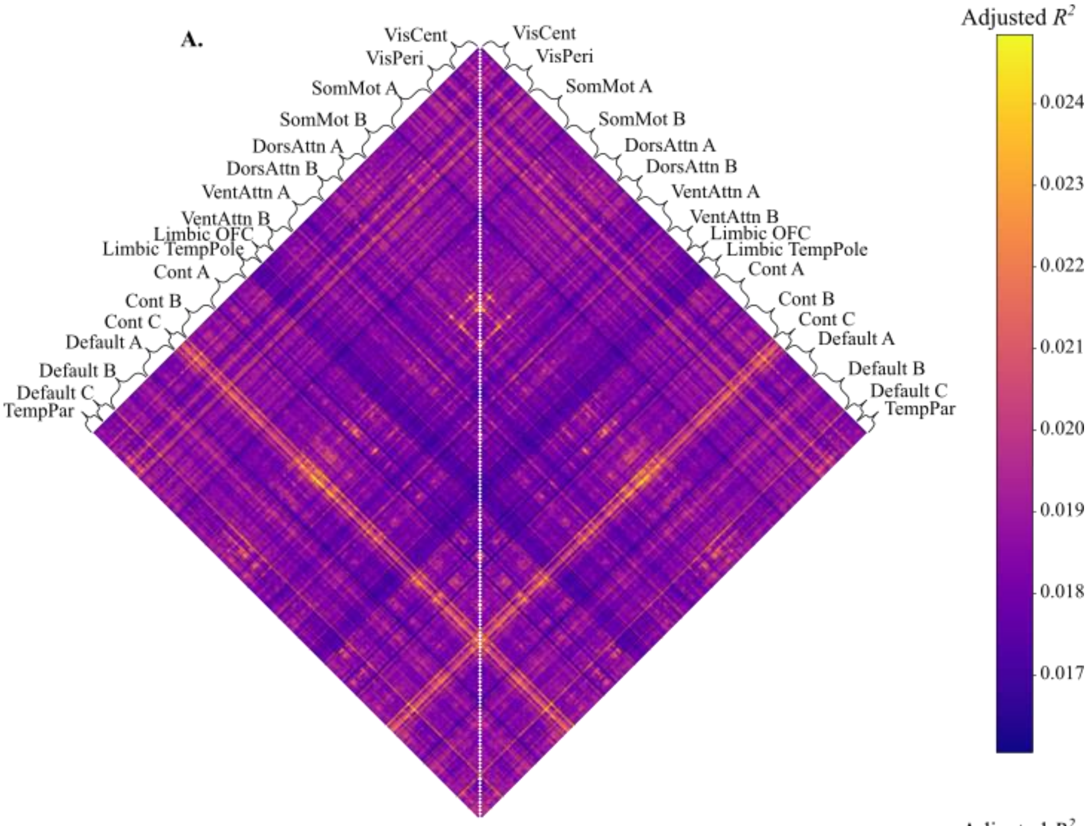
VisCent
VisPeri
SomMot A
SomMot B
DorsAttn A
DorsAttn B
VentAttn A
VentAttn B
Limbic OFC
Limbic TempPole
Cont A
Cont B
Cont C
Default A
Default B
Default C
TempPar
Adjusted R^2
0.024
0.023
0.022
0.021
0.020
0.019
0.018
0.017

B.

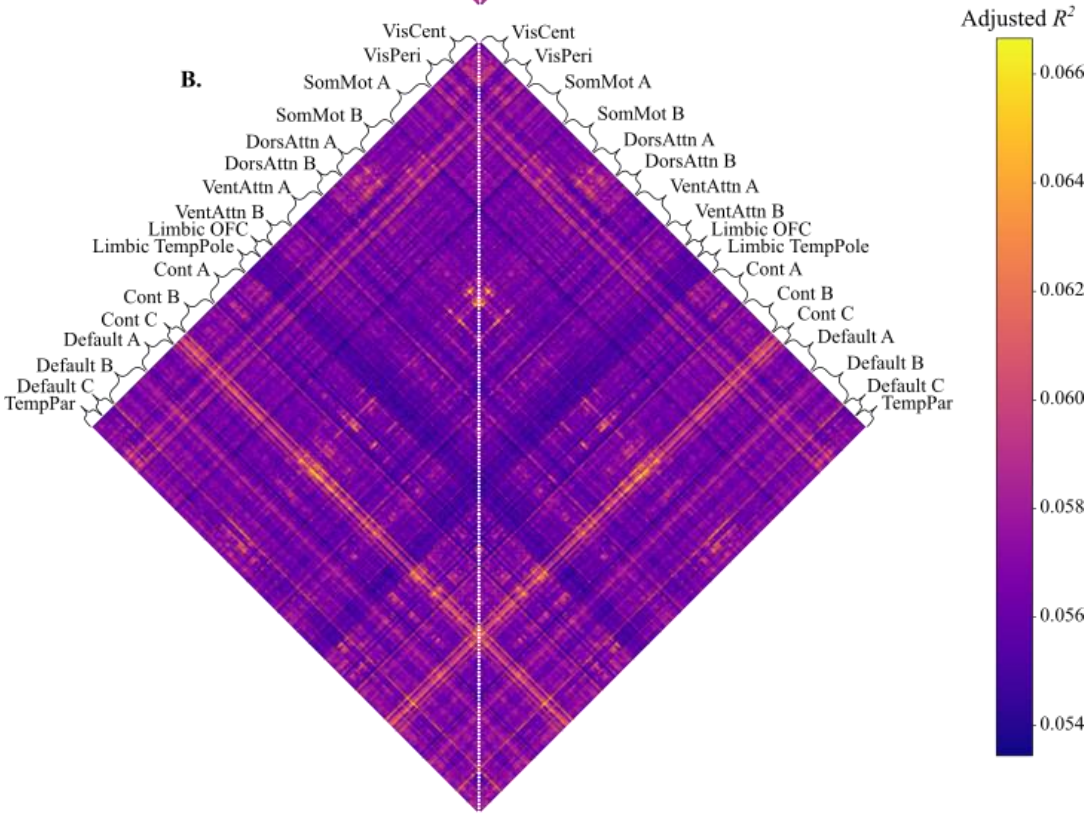
VisCent
VisPeri
SomMot A
SomMot B
DorsAttn A
DorsAttn B
VentAttn A
VentAttn B
Limbic OFC
Limbic TempPole
Cont A
Cont B
Cont C
Default A
Default B
Default C
TempPar
Adjusted R^2
0.066
0.064
0.062
0.060
0.058
0.056
0.054

Supplementary Figure 1. Comparison of adjusted R^2 values between prewhitened (A.) and non-prewhitened (B.) analysis pipelines. Presented values are average R^2 values from all individual subject models at each edge.

Supplementary References

Avants, B. B., Epstein, C. L., Grossman, M., & Gee, J. C. (2008). Symmetric diffeomorphic image registration with cross-correlation: evaluating automated labeling of elderly and neurodegenerative brain. *Medical image analysis*, *12*(1), 26-41.

Ciric, R., Rosen, A. F., Erus, G., Cieslak, M., Adebimpe, A., Cook, P. A., Bassett, D. S., Davatzikos, C., Wolf, D. H., & Satterthwaite, T. D. (2018). Mitigating head motion artifact in functional connectivity MRI. *Nature protocols*, *13*(12), 2801-2826.

Ciric, R., Thompson, W. H., Lorenz, R., Goncalves, M., MacNicol, E. E., Markiewicz, C. J., Halchenko, Y. O., Ghosh, S. S., Gorgolewski, K. J., & Poldrack, R. A. (2022). TemplateFlow: FAIR-sharing of multi-scale, multi-species brain models. *Nature methods*, *19*(12), 1568-1571.

Dale, A. M., Fischl, B., & Sereno, M. I. (1999). Cortical surface-based analysis: I. Segmentation and surface reconstruction. *NeuroImage*, *9*(2), 179-194.

Evans, A. C., Janke, A. L., Collins, D. L., & Baillet, S. (2012). Brain templates and atlases. *NeuroImage*, *62*(2), 911-922.

Greve, D. N., & Fischl, B. (2009). Accurate and robust brain image alignment using boundary-based registration. *NeuroImage*, *48*(1), 63-72.

Jenkinson, M., Bannister, P., Brady, M., & Smith, S. (2002). Improved optimization for the robust and accurate linear registration and motion correction of brain images. *NeuroImage*, *17*(2), 825-841.

Klein, A., Ghosh, S. S., Bao, F. S., Giard, J., Häme, Y., Stavsky, E., Lee, N., Rossa, B., Reuter, M., & Chaibub Neto, E. (2017). Mindboggling morphometry of human brains. *PLoS computational biology*, *13*(2), e1005350.

Satterthwaite, T. D., Elliott, M. A., Gerraty, R. T., Ruparel, K., Loughead, J., Calkins, M. E., Eickhoff, S. B., Hakonarson, H., Gur, R. C., & Gur, R. E. (2013). An improved framework for confound regression and filtering for control of motion artifact in the preprocessing of resting-state functional connectivity data. *NeuroImage*, *64*, 240-256.

Schaefer, A., Kong, R., Gordon, E. M., Laumann, T. O., Zuo, X.-N., Holmes, A. J., Eickhoff, S. B., & Yeo, B. T. (2018). Local-global parcellation of the human cerebral cortex from intrinsic functional connectivity MRI. *Cerebral cortex*, *28*(9), 3095-3114.

Smith, S. M., Jenkinson, M., Woolrich, M. W., Beckmann, C. F., Behrens, T. E., Johansen-Berg, H., Bannister, P. R., De Luca, M., Drobnjak, I., & Flitney, D. E. (2004). Advances in functional and structural MR image analysis and implementation as FSL. *NeuroImage*, *23*, S208-S219.

Tustison, N. J., Avants, B. B., Cook, P. A., Zheng, Y., Egan, A., Yushkevich, P. A., & Gee, J. C. (2010). N4ITK: improved N3 bias correction. *IEEE transactions on medical imaging*, *29*(6), 1310-1320.

Zhang, Y., Brady, M., & Smith, S. (2001). Segmentation of brain MR images through a hidden Markov random field model and the expectation-maximization algorithm. *IEEE transactions on medical imaging*, *20*(1), 45-57.
